# Flow Cytometry and Fecal Indicator Bacteria Analyses for Fingerprinting Microbial Pollution in Karst Aquifer Systems

**DOI:** 10.1029/2021WR029840

**Published:** 2022-04-27

**Authors:** Luka Vucinic, David O’Connell, Rui Teixeira, Catherine Coxon, Laurence Gill

**Affiliations:** ^1^ Department of Civil, Structural and Environmental Engineering University of Dublin Trinity College Dublin Ireland; ^2^ Department of Geology and Trinity Centre for the Environment University of Dublin Trinity College Dublin Ireland

**Keywords:** karst, flow cytometry, microbial, spring, turbidity

## Abstract

Microbial pollution of aquifers is a persistent water quality problem globally which poses significant risks to public health. Karst aquifer systems are exceptionally vulnerable to pollution from fecal contamination sources as a result of rapid recharge of water from the surface via discrete pathways linked to highly conductive, solutionally enlarged conduits alongside strong aquifer heterogeneity. Consequently, rapid changes in microbial water quality, which are difficult to monitor with expensive and time‐consuming conventional microbiological methods, are a major concern in karst environments. This study examined flow cytometric (FCM) fingerprinting of bacterial cells in groundwater together with fecal indicator bacteria (FIB) at nine separate karst springs of varying catchment size over a 14 month period in order to assess whether such a technique can provide faster and more descriptive information about microbial pollution through such karst aquifer systems. Moreover, the data have also been evaluated with respect to the potential of using turbidity as an easy‐to‐measure proxy indicator of microbial pollution in a novel way. We argue that FCM provides additional data from which enhanced insights into fecal pollution sources and its fate and transport in such karst catchments can be gained. We also present valuable new information on the potential and limitations of turbidity as an indicator of fecal groundwater contamination in karst. FCM has the potential to become a more widely used tool in the field of contaminant hydrogeology.

## Key Points

- Flow cytometry can provide enhanced insights into the fate and transport of fecal pollution in karst aquifers
- Stable bacterial communities in karst aquifers can limit the potential of flow cytometry to predict changes in fecal indicator bacteria
- Enterococci should be analyzed with *Escherichia* *coli* if turbidity is to be evaluated as an inexpensive groundwater quality indicator in karst springs

## 1 Introduction

Contamination of groundwater by pathogenic bacteria, protozoa, and viruses of fecal origin has been associated with waterborne disease outbreaks across various hydrogeological environments worldwide. Significantly, the extent of such microbiological contamination and transport of waterborne pathogens in aquifers is still not well understood (Bradford & Harvey, 2017; Cronin & Pedley, 2002). Generally, groundwater is considered to be less vulnerable than surface water to microbial pathogenic contamination from fecal matter, however, polluted groundwater is still responsible for a disproportionate fraction of reported waterborne disease outbreaks, particularly in developing countries and rural regions (Bradford & Harvey, 2017; Buckerfield et al., 2019; Jin & Flury, 2002; WHO & UNICEF, 2014). Furthermore, this problem is shared in developed countries such as the United States where between 750,000 and 6 million illnesses per year have been attributed to contaminated groundwater (Macler & Merkle, 2000; Reynolds et al., 2008). Serious cases of pathogenic bacterial contamination of aquifers have been reported following episodic heavy rain events, a notable example occurring at Walkerton (Ontario, Canada) in May 2000, where the rapid transport of pathogenic contaminants through a highly fractured carbonate aquifer system to water supply wells resulted in 2,300 illnesses and 7 deaths (O’Connor, 2002; Worthington & Smart, 2017). Many incidences of waterborne diseases can be associated with contamination of surface water and groundwater due to failing on‐site domestic wastewater treatment systems (DWTSs), spreading of agricultural fecal matter (i.e., manure) and other farming activities in rural and less urbanized areas (Fetter, 2001; WHO, 2003). Despite a decrease in recent decades in waterborne disease outbreak risks related to municipal water supply sources, no corresponding decrease in disease outbreak risks for untreated or inadequately treated groundwater has been observed on the basis of outbreak reports (Craun, 2012). Hence, microbial pathogenic contamination of aquifers remains an ongoing, globally important, water quality problem (Ashbolt, 2004; Nguyen et al., 2016). Moreover, as a result of increasing numbers of point and nonpoint sources of fecal pollution in catchments (Barrett et al., 1999; Cronin & Pedley, 2002; Misstear et al., 1996), population growth, extreme weather events associated with climate change, and rapid land‐use alterations, it should be expected that such water quality problems will be exacerbated in the future (Bradford & Harvey, 2017; Delpla & Rodriguez, 2014; Pratt & Chang, 2012; Sliva & Williams, 2001). Therefore, it is of utmost importance that enhanced understanding is developed about the potential factors affecting microbial groundwater quality, the associated pathogen subsurface transport mechanisms in various hydrogeological systems, and the pollution impacts of disease‐causing microorganisms on water quality at springs, boreholes, and wells (Savio et al., 2018).

Groundwater from karst aquifers, through springs, boreholes, and wells, is a major source of drinking water which has recently been estimated to supply at least 9% of the world's population (Stevanović, 2019). In many countries and regions (e.g., Montenegro and other countries within the Dinaric region of Europe, Austria, southwest China, etc.), karst water contributes 50% or more to regional freshwater supplies (Hartmann et al., 2014; Wu et al., 2009) and to the water supply of large cities such as San Antonio, Vienna, Rome, and Damascus (Chen et al., 2017; Kresic & Stevanovic, 2010). Karst aquifers are particularly vulnerable to contamination from a variety of different sources. Such vulnerability is due to relatively fast recharge of water from the surface into the upper part of the karst system (epikarst), because of commonly thin soil coverage with a low field capacity, and/or the rapid infiltration into the groundwater system directly through discrete flowpaths (i.e., swallow holes [also known as “ponors”] and closed depressions [dolines]), which connect directly into the highly conductive network of solutionally enlarged conduits where turbulent flow conditions generally dominate, which finally discharge at springs (Gutiérrez & Gutiérrez, 2016; Hillebrand et al., 2012; Thorn & Coxon, 1992; Vesper et al., 2001; White & White, 2005). For example, Ender et al. (2018) have shown that microbial contamination of springs connected to swallow holes is systematically higher than for springs not connected to a swallow hole. These additional threats to karst groundwater contamination through concentrated inputs from fecal point and nonpoint sources have been regularly observed worldwide (Coxon, 2011; Heinz et al., 2009; Kaçaroğlu, 1999). However, under certain scenarios, even in karst regions, it is possible that some of the water will reach the karst aquifer several months after a precipitation event as it might be stored for some time in the subsoil and the epikarstic zone (Ford & Williams, 2007). This is more common in areas covered by thick low permeability soils and subsoils deposited on top of the karstified rocks. Nevertheless, high connectivity of surface and groundwater systems exists globally in almost all karst environments, as well as high transmissivity and connectivity of aquifers over large areas (Hartmann et al., 2014). Such connectivity enables water contaminated with viable and culturable pathogenic microorganisms to reach water sources used for domestic and irrigation purposes. Consequently, the resulting rapid changes in water quality in karst environments due to aquifer heterogeneity and a variety of pollution sources are a major concern for water resource managers and stakeholders (Auckenthaler et al., 2002; Butscher et al., 2011; Drew, 1996; Reischer et al., 2011; Ryan & Meiman, 1996). Springs offer appropriate natural locations for monitoring pollutant concentration dynamics in karst aquifer systems as they provide an integrated picture of contaminant transport through a karst conduit network, compared to wells and boreholes which are not necessarily directly connected to the most transmissive parts of the aquifer (Geyer et al., 2007).

Despite previous studies focused on studying microbial contaminants in groundwater, including karstified aquifers (Vesper et al., 2001), significant knowledge gaps exist with respect to the survival of pathogenic organisms compared to standard fecal indicator bacteria (FIB) in the subsurface and groundwater. This is of obvious concern as the microbial examination of water using FIB is often the only method used to determine the sanitary quality of water and to assess the potential public‐health risks from waterborne diseases (Francy et al., 2000). In addition, conventional microbiological methods for complete screening of microorganisms in water are still very expensive, complicated, labor‐intensive, and time‐consuming (NRC, 2004; Nemati et al., 2016). Standard methods for culturing bacteria are relatively simple and regarded as a low‐cost techniques, but they are limited by low sensitivity, as well as being labor intensive with a high time commitment (usually taking between 24 and 48 hr) to produce results. In addition, such methods may provide false negatives because fecal coliform bacteria such as *Escherichia coli* often exist in a viable but nonculturable (VBNC) state (Ramírez‐Castillo et al., 2015). The use of turbidity as an easy‐to‐measure indicator of microbial water contamination has been explored and even though some significant correlations were found between turbidity and FIB (*E. coli*, etc.), systematic relationships between turbidity and FIB counts in karst springs seem to be highly site‐specific (Allen et al., 2008; Huey & Meyer, 2010; Pronk et al., 2006, 2007; Schiperski, 2018). Accordingly, its use to predict periods of elevated microbial pathogenic contamination has limited applications (Allen et al., 2008; Schiperski, 2018). In a similar way, the suitability of dissolved organic carbon (DOC) and/or total organic carbon (TOC) as indicators of bacterial contamination have been studied in the past in karst environments (Heinz et al., 2009; Thurman, 1985). However, it has been concluded that these parameters are even less indicative of bacterial contamination than turbidity (Heinz et al., 2009) or turbidity in combination with TOC (Pronk et al., 2006). Moreover, several other techniques have been assessed as indicators of fecal pollution in karst aquifer systems, such as particle‐size distribution measurements (Pronk et al., 2007), measurements of enzymatic activity of *E. coli* (Ender et al., 2017), and tryptophan‐like fluorescence measurements (Frank et al., 2018). In contrast, another tracing technique known as microbial source tracking (MST), for quantification of microbes based on their characteristic DNA fragments by performing DNA extraction and quantitative real‐time polymerase chain reaction (qPCR) analysis, has been used to assess the potential health risks associated with environmental waters contaminated with fecal microorganisms and viruses (Ahmed et al., 2014; Gill, Babechuk, et al., 2018; Gill, O’Flaherty, et al., 2018; Savio et al., 2018). Unfortunately, MST approaches are too complex, expensive, and time‐consuming to be performed routinely at present, and usually do not distinguish between viable and nonviable microorganisms. Such limitations diminish their value in assessing the efficacy, for example, of disinfection processes or rapid adaptation of water abstraction to reduce associated risks, as well as for observing the survival rates and fate of fecal bacteria during their transport in the subsurface under different flow conditions (Safford & Bischel, 2019; Tryland et al., 2015).

Flow cytometry (FCM) is a method, first developed in the 1960s, that is used routinely in biology and medicine to classify small particles suspended in solution such as cells or microspheres (Büscher, 2019; Coggins et al., 2020; Sack et al., 2006). Significantly, FCM is emerging as a very promising fluorescence‐based and cultivation‐independent technique in environmental microbiology as a result of the ability of flow cytometers to rapidly quantitate bacteria and discriminate them from debris by staining the bacterial DNA with fluorescent dyes (Gatza et al., 2013; Prest et al., 2013). Safford and Bischel (2019) have identified and examined nearly 300 studies from the last two decades mainly focused on FCM applications in water treatment, distribution, and reuse, but conclude that more research is needed in order to realize the full potential of FCM. Moreover, although extensive research has been carried out using FCM on the monitoring and assessment of drinking water quality (e.g., Hammes et al., 2008; De Roy et al., 2012; Besmer et al., 2014; Prest et al., 2016; Van Nevel et al., 2017) and characterization of microbial communities (e.g., Hammes & Egli, 2010; Van Nevel et al., 2013), studies in karst aquifer systems are rare and so far have mainly appear to have been conducted with a single most commonly used FCM parameter (Total cell count [TCC]; e.g., Besmer et al., 2017; Page et al., 2017; Sinreich et al., 2014). There has been little investigation into whether other FCM parameters (intact cell count [ICC], high nucleic acid [HNA], and low nucleic acid [LNA] counts) can be useful in terms of enabling a better understanding the fate and transport of microbial pollutants and/or for modeling studies. Furthermore, a range of FCM parameters have never been correlated with groundwater turbidity since such research to date has tended to focus on FIB rather than FCM fingerprinting.

The objectives of this study, therefore, are to: (a) assess the use of flow cytometry as a method for evaluating fecal indicator bacteria at karst springs, (b) assess whether there are any additional benefits of evaluating FCM parameters (ICC, HNA, and LNA counts) over just using TCC, (c) compare the microbial contamination of a range of lowland karst springs in the west of Ireland, and (d) use flow cytometry and parallel fecal indicator bacteria results to reevaluate turbidity as an appropriate cost effective, real‐time indicator of microbial contamination of karst springs.

## 2 Materials and Methods

### 2.1 Study Sites

**Figure 1:**
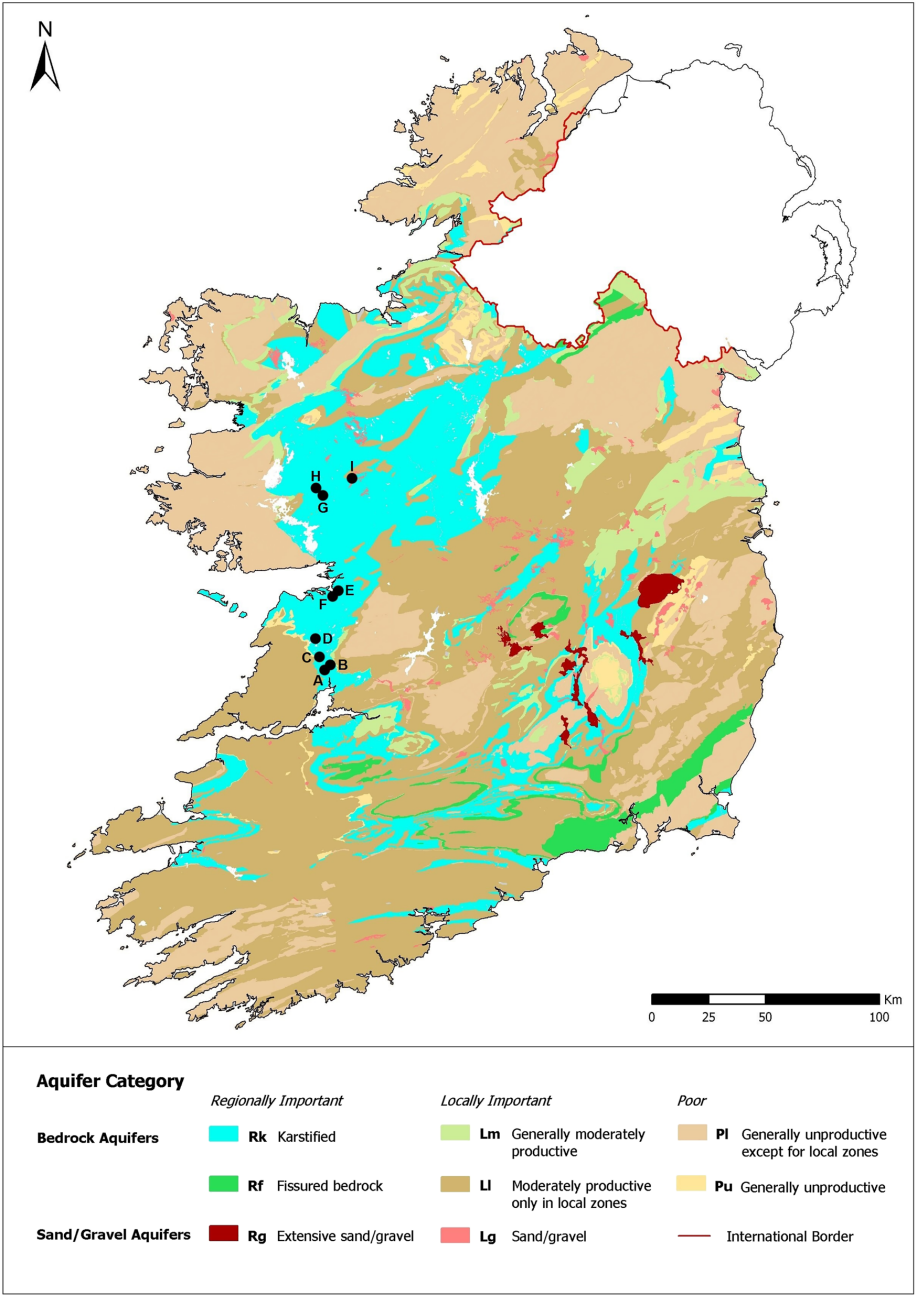
National aquifer classification map of the Republic of Ireland (GSI, 2020) with locations of nine karst springs from this study.

Nine springs from separate karst catchments in the west of Ireland (A to I) were selected for this study: A, B, C, and D are springs located in County Clare, E and F are intertidal springs located in County Galway, and G, H, and I are springs located in County Mayo (see Figure 1). Selection criteria included a representative coverage of lowland karst catchments in Ireland in terms of catchment size, dominant groundwater vulnerabilities within catchments and karst system types, densities of on‐site domestic wastewater systems (DWTSs) and land‐use settings. Detailed information for the selected catchments is provided in Table 1.

**Table 1:** Site‐Specific Information for Catchments

Site	ZOC^a^ (km^2^)	Groundwater vulnerability^f^ ^,^ ^h^	Karst system^f^	Bedrock^f^	Soils^a^	Subsoils^a^	Land use^a^	DWTSs density^g^ (no/km^2^)	DWK200^g^ (%)
A	63.6	Ex and High	Rkc	DPBL and NSS	BminDW, BminSW, AminPD	KaRck, TLs, TNSSs	PG, AR, MF, NV	11.13	4.52
B	427.9	Ex and High	Rkc	DPBL with NSS and DORS	BminSW, BminDW, TNSSs	KaRck, TLs	PG, AR, MF, NV	5.41	3.02
C	11.9^b^	Ex, High, and Mod	Rkc	DPBL and NSS	BminDW, AminPD, BminSW	KaRck, TNSSs	PG, AR, MF, NV	7.36	5.68
D	131.1	Ex	Rkc	DPBL	BminSW	KaRck	PG, AR, MF, NV	2.99	10.46
E	88^c^	Ex, High, and Mod	Rkc	DPBL	BminDW	TLs	PG	9.83	3.68
F	498^c^	Ex, High, Mod, and Low	Rkc	DPBL and DORS	BminSW, BminDW	TLs, TDSs	PG, MF	4.27	3.79
G	1.74	High and Mod	Rkc	DPBL	BminSW, BminDW	TLs, GLs	PG	7.47	7.69
H	31.9^d^	Ex and High	Rkc	DPBL	BminDW	TLs	PG	9.52	8.75
I	3.4^e^	High, Mod, and Low	Rkd/Rkc^e^	DPBL	BminDW	TLs	PG	12.94	0

*Note*. AminPD, Mineral poorly drained soils; AR, Arable land; BminDW, Deep well drained mineral soils; BminSW, Shallow well‐drained mineral soils; DORS, Devonian Old Red Sandstones; DPBL, Dinantian Pure Bedded Limestone; DWK200, Percentage of DWTSs in the catchment that are within 200 m of at least one karst feature such as swallow hole, estavelle, or turlough; Ex, Extreme vulnerability; GLs, Carboniferous Limestone Sands and Gravels; High, High vulnerability; KaRck, Bedrock at or close to surface; Low, Low vulnerability; MF, Mixed Forest; Mod, Moderate vulnerability; NSS, Namurian Sandstones; NV, Open spaces with little or no vegetation; PG, Pasture/grassland; Rkc, Conduit flow‐dominated karst aquifer; Rkd, Diffuse flow dominated karst aquifer; TDSs, Devonian Sandstone Tills ‐ diamictons; TLs, Carboniferous Limestone Tills ‐ diamictons; TNSSs, Namurian Shales and Sandstone Tills; ZOC, Zone of Contribution.

^a^ From EPA Ireland (2020)—except ^b, c, d and e^.

^b^ From Morrissey (2013).

^c^ Delineated using information provided in McCormack et al. (2014) and Morrissey et al. (2020).

^d^ Delineated using information provided in Murphy et al. (2015).

^e^ From Schuler et al. (2021).

^f^ From Geological Survey of Ireland (GSI) (2020)—except ^e^.

^g^ Calculated using ArcGIS; DWTSs layers obtained from the EPA Ireland (GeoDirectory) under the same conditions as explained in Gill and Mockler (2016); karst features data from Geological Survey of Ireland (GSI; 2020).

^h^ The concept of Groundwater Vulnerability in Ireland has been used mainly on the basis of the thickness and permeability of the subsoils overlying the bedrock aquifer (see DoELG/EPA/GSI (1999) for details).

### 2.2 On‐Site Measurements, Sampling Procedure, and Frequency

Water samples were collected for microbiological analysis on a monthly basis from each spring from September 2017 until November 2018 (with the exception of October 2017), in order to provide data across a range of meteorological and flow conditions. All samples were collected directly at the karst spring outlets using sterile bottles and kept in a cool box boxes during the transfer from the field to the laboratory. Upon arrival in the laboratory, samples were analyzed within an hour to limit changes in bacterial counts as much as possible. In addition to the analysis of microbial indicators in the laboratory, physico‐chemical parameters of water (pH, temperature, turbidity, and EC) were measured on‐site at the time of sample collection with a HI9829 multiparameter meter (Hanna Instruments) which was calibrated (according to the manufacturer's instructions) prior to each field sampling campaign. Rainfall data (see Supporting Information S1) has been obtained from the Irish Meteorological Service (Met Eireann) and effective rainfall has been calculated according to methodology described in Schulte et al. (2005).

### 2.3 Laboratory Methods and Microbiological Analysis

Microbiological analysis included FCM microbial fingerprinting (TCC, ICC, LNA, and HNA bacterial counts) and quantification of total coliforms (TC) and FIB (*E. coli* and enterococci). Measurement of total cell count (TCC) with FCM refers to quantification of the total microbial cell density (all live, damaged, and dead bacterial cells per 1 ml of sample), while measurement of intact cell count (ICC) with FCM refers to quantification of only live bacterial cells with intact membranes in the same karst groundwater spring samples. Moreover, measurement of HNA and LNA bacterial communities with FCM refers to bacteria within the ICC group on the basis of their DNA content (high and low).

For FCM microbial fingerprinting, samples were pre‐treated and diluted in physiologic phosphate‐buffered saline containing 0.2% Pluronic^®^ F68 and 1 mmol/L EDTA (which has been previously passed through a 0.22 μm syringe filter). In the next step, the Eawag (Swiss Federal Institute of Aquatic Science and Technology) and BD Biosciences staining protocol for rapid counting of live and dead bacteria by using BD™ Cell Viability Kit (Cat. No.: 349,483) staining dyes was followed, whereby 5 μL of propidium iodide and 5 μL of thiazole orange permanent dyes were added into each tube with 500 μL of pre‐treated sample suspension. Thiazole orange, the parent compound of SYBR stain family, stains all cells and enables discrimination of cells from debris, while propidium iodide is impermeable to live cells with intact membranes and stains only damaged and dead cells with compromised membranes. The final concentrations of staining dye solutions in all sample tubes were 420 nmol/L for thiazole orange and 43 μmol/L for propidium iodide. All tubes with individual samples were capped, vortexed for 30 s and incubated (37°C) in the dark for 10 min. Samples were analyzed at the Flow Cytometry Facility (the School of Biochemistry and Immunology) at Trinity College Dublin Biomedical Sciences Institute using a BD Accuri C6^®^ flow cytometer, and a software analysis template, with predefined workspace, instrument settings, fixed gates and parameters, developed by researchers at Eawag as described in detail in Gatza et al. (2013). All measurements for FCM fingerprinting were performed in triplicate for quality control and statistical analysis with a constant (medium) flow rate of the instrument during data acquisition process in order to achieve comparable data.

Aseptic techniques were employed for each microbiological analysis performed at the Environmental Engineering Department laboratory at Trinity College Dublin for the presence, absence, and the most probable number (MPN) of colony forming units (CFU) per 100 mL of total coliforms (TC), *E. coli*, and enterococci. These analyses were carried out using IDEXX Colilert‐18 (ISO 9308‐2) and IDEXX Enterolert‐E (ISO 7899‐1) test kits in conjunction with the IDEXX Quanti‐Tray/2000 and IDEXX Quanti‐Tray Sealer. Each individual quanti‐tray with diluted sample mixed with an appropriate powder from the test kits was incubated at 35°C for 18 hr for TC and *E. coli* analysis, and at 41°C for 24 hr for enterococci analysis. After incubation, the number of large and small wells that were positive (based on a color change and/or UV fluorescence) were counted and these counts were converted into results (MPN and ±95% confidence intervals) using IDEXX MPN Generator 1.4.4 software. For quality control, every month at the time of the analysis a few samples were randomly selected and analyzed in duplicate or triplicate.

## 3 Results and Discussion

### 3.1 Flow Cytometry—Total Cell Counts

**Figure 2:**
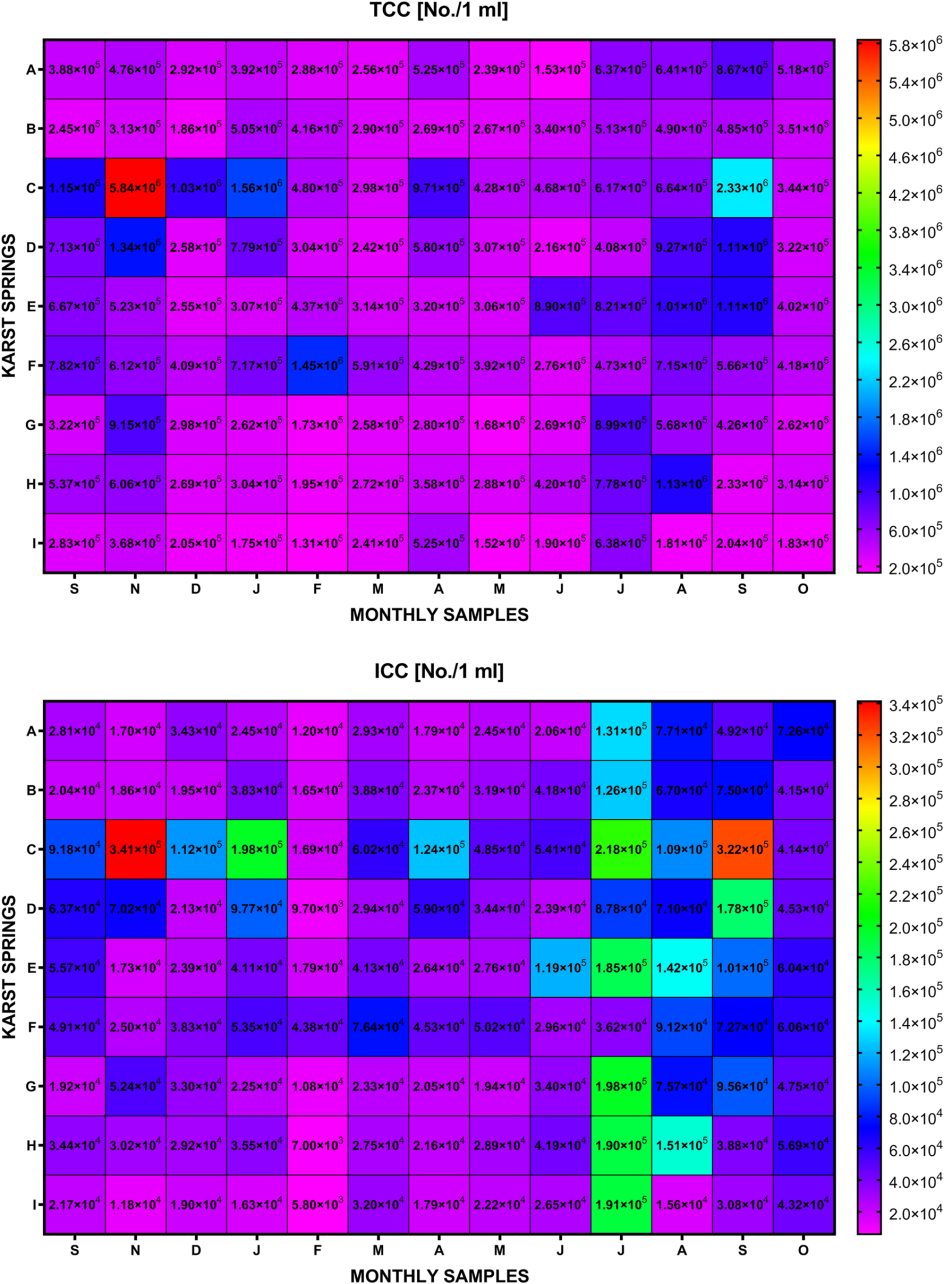
FCM fingerprinting: dynamics of TCC and ICC abundances at karst springs.

The overall FCM bacterial counting results, as shown in Figure 2 (and in Figure S1 in Supporting Information S1), were used to examine changes in concentrations of bacterial cells over time at individual karst springs, and to analyze observed differences between selected karst springs. In this study, TCC values for most springs were measured consistently at a general level of 10^5^ per mL with only Spring C showing TCC abundances in at levels of 10^6^ cells per mL (the highest recorded in this study) throughout the entire sampling period. These TCC results are generally in agreement with the findings of Sinreich et al. (2014) which showed a high degree of uniformity at individual karst springs in Switzerland, but variability from one spring to another. Furthermore, total microbial cell densities (TCC) recorded at karst springs in this study were in a similar range of TCC values observed at the representative (highly vulnerable) karst springs in Switzerland (Sinreich et al., 2014). Importantly, the relatively consistent TCC concentrations over time (despite some occasional notable spikes) as observed at all the karst springs in this study, further support numerous scientific findings that a stable microbial community exists in the karst subsurface (Farnleitner et al., 2005; Pronk et al., 2006, 2009; Sinreich et al., 2014). An implication of this is the hypothesis presented by Sinreich et al. (2014) that total bacterial densities are primarily determined by the long‐term aquifer and catchment characteristics, and less by the short‐term contamination events and/or hydrological factors. Therefore, TCC fluctuations over time at karst springs as a sole FCM parameter may provide some interesting information regarding the karst aquifer systems and their dynamics, but only limited information in terms of the fate and transport dynamics of microbial fecal pollution through karst systems. Consequently, a more comprehensive approach must be taken in order to fully evaluate the potential benefits of FCM for addressing relevant contaminant hydrogeology issues in karst environments.

Although TCC values at individual karst springs in this study were measured mostly within the same order of magnitude, fluctuations over time have been observed at all nine monitored karst springs, as shown in Figure 2 (and Figure S1 in Supporting Information S1). Springs A and B from catchments of very different sizes (Catchment B is more than six times larger than Catchment A) are located very close to each other in County Clare and both catchments have similar groundwater vulnerabilities (predominantly Extreme and High), bedrock geology, land use, and relatively similar soils and subsoils. They contain different DWTS densities with Catchment A at twice the density compared with Catchment B, as well as having a higher percentage of domestic wastewater treatment systems within 200 m of karst features (e.g., swallow holes, estavelles, and turloughs) that can provide rapid transport of contaminants into karst aquifer systems (DWK200). Hence, it is not surprising that, in general, somewhat higher TCC concentrations were recorded at Spring A than at Spring B. However, both springs have shown fairly similar TCC fluctuations over time, such as similar TCC concentration increases between July and October 2018. A comparison with rainfall across the sampling period (see Supporting Information S1, Figures S2a, S2b and Table S3 in Supporting Information S1) shows the highest correlation between TCC counts at Spring A with rainfall that occurred 2 days prior to sampling (*r* = 0.41) compared to the much larger Catchment B that shows the best correlation with rainfall that fell 5 days prior to sampling (*r* = 0.46), reflecting the longer transport times.

The other two springs located in County Clare, Springs C and D, had the largest concentrations of TCC recorded in November 2017, which corresponded with a high precipitation period in the west of Ireland (see Figures S2c, S2d in Supporting Information S1) as well as other multiple spikes throughout the entire monitoring period. Yet, even though that karst system type, dominant bedrock and surficial geology, groundwater vulnerabilities and land use in both Catchments C and D are similar, the higher TCC concentrations picked up in Spring C is likely to be a result of significant differences in sizes of catchment areas (Catchment C is approximately 11 times smaller than Catchment D), in conjunction with the differences in DWTSs densities—Catchment C has a much higher DWTS density than Catchment D. Catchment D, however, does have a much higher DWK200 compared to Catchment C (the highest across all the springs in fact), which may be causing the high variations of TCC values at the spring linked to rapid movement of contaminants into the highly transmissive karst network under enhanced hydrological conditions. The correlation with rainfall for these two catchments (see Table S3 in Supporting Information S1) shows the highest correlation between TCC counts at Spring C with rainfall that occurred on the day of sampling (*r* = 0.79), suggesting a very fast reaction in such a small catchment, as expected. For the much larger Catchment D, although it does show the highest correlation with rainfall that fell 2 days prior to sampling (*r* = 0.58) reflecting the longer transport times, an almost equally high correlation is revealed for rainfall that fell on the day of sampling (*r* = 0.56), which may be indicative of the highly transmissive pathways for contaminant transport.

The two intertidal karst springs (E and F) are from adjacent catchments in County Galway ‐the first a smaller autogenic recharged catchment, the second a much larger conduit‐dominated allogenic recharged catchment with significant groundwater‐surface water interaction in the winter time—see Morrissey et al. (2020). Spring E had noticeably elevated TCC during the summer months in 2018 despite the long period in the summer without any effective recharge to the aquifer which also caused more prolonged saltwater intrusion impacts (as confirmed by EC data in Supporting Information S1). This may be reflective of a more constant pollutant load from DWTSs directly into the karst network which are present at a higher density compared to the catchment for Spring F which did not show such an increase in the summer but rather a very notable increase in TCC in February 2018. This particular catchment is characterized by extensive groundwater flooding in the winter time in the form of intermittent lakes that form known as turloughs. These act to hydraulically damp the rainfall‐discharge relationship for flows from the main spring during these flood period, but do also provide a direct pathway for surface contaminants into the groundwater over a wide area. The correlation with rainfall for these catchments (see Table S3 in Supporting Information S1) shows a weak correlation between TCC counts with rainfall that occurred 2 days (*r* = 0.24) prior to sampling at Spring E (the smaller catchment, which is similar in size to Catchment A), compared to correlations with rainfall that fell 4 and 5 days prior to sampling (*r* = 0.26) for the much larger catchment for Spring F, as would be expected.

Springs G, H, and I emerge in County Mayo, in predominantly agricultural catchments (pastures/grasslands) with relatively high densities of DWTSs (Table 1). At these three springs, elevated TCC values have been observed during the summer 2018 (which again was a long period of no effective rainfall as shown in Figure S2g, h, j in Supporting Information S1) and so these higher values may be revealing more direct infiltration of DWTS effluent into the karst network which would be more pronounced at low flow, aligned to the proximity of DWTSs near karst features in Catchments G and H. High TCC values were also picked up in November 2017 in Spring G and to a lesser extent Spring H which coincided with a high rainfall event. The correlation with rainfall for these catchments (see Table S3 in Supporting Information S1) shows a weak correlation between TCC counts with rainfall that occurred on the day of sampling (*r* = 0.34) at Spring G (a very small catchment of just 1.7 km^2^), compared to Spring H which revealed the strongest correlation with rainfall that fell 2 days prior to sampling (*r* = 0.45) for the larger catchment of 31.9 km^2^. Spring I revealed more consistent TCC results across the year with some slight elevated values during periods of zero effective rainfall in the summer. This small catchment (3.4 km^2^) has no visible karst features of interest at the surface, and recent hydrological studies of the catchment have shown that under moderate recharge conditions the aquifer has a damped response to rainfall, but during higher sustained recharge conditions the spring becomes directly connected to a nearby river via a high level conduit, providing a much faster response to rainfall events (Schuler, 2020; Schuler et al., 2021).

In general, the TCC results reveal interesting comparisons between catchments. It could be argued that large increases in total microbial cell densities at certain times which are not being observed at nearby springs with similar TCC patterns may be a result of isolated fecal contamination events rather than more system‐dependent factors (e.g., infiltration of cell‐rich water in the system as a result of localized precipitation event, enriched biocenoses [i.e., environmental organisms], etc.). However, there are several possible explanations for any significant rise in total microbial cell densities at karst springs, therefore, TCC data should be interpreted with caution. Hence, due to the clear limitations of relying on a sole FCM‐TCC parameter, other FCM parameters were evaluated in terms of their applicability to provide a better understanding of microbial pollution of karst aquifer systems.

### 3.2 Flow Cytometry—Numbers and Types of Live Bacteria (ICC, LNA, and HNA)

Temporal changes in the numbers and percentages of live bacteria with intact membranes (ICC) at springs are presented in Figure 2. These data show that elevated ICC concentrations were mainly recorded during the summer months during low flow conditions at the karst springs. Furthermore, although some elevated ICC concentrations were also observed in other, more hydrologically active seasons at some springs (e.g., Spring C), these spikes were much less significant than those recorded during the summer months. The significance of any ICC spike can be further investigated by looking at the percentages of ICC within the total microbial cell densities. The observed higher survival rates of bacteria during the summer months may be attributed to more elevated groundwater temperatures (see Figure 6 for groundwater temperature data) coupled with less dispersion, possible persistent plumes of contaminants from DWTSs, and/or certain agricultural activities. Additionally, the ICC values are mainly in the same order of magnitude as TCC values for some springs (D, E, G, H, and I) but not for other springs (A, B, C, and F). The different orders of magnitude in catchments B and F could be attributed to the longer average pathways for bacterial transport associated with their large ZOCs. This could be a possible explanation for the catchment A as well, even though it is notably smaller than catchments B and F. However, catchment C is very small (11.9 km^2^), and so differences in the orders of magnitude of TCC and ICC values could not be attributed to such longer average pathways for the bacterial transport through the karst aquifer. A possible explanation for this might be that a portion of bacterial loading into this system consists of enteric bacteria that died before entering karst aquifer system (as a result, e.g., of well‐designed and maintained on‐site domestic wastewater treatment systems). Moreover, the TCC and ICC patterns recorded at Spring C suggest that another, much more significant, portion of bacterial loading could be relatively close to the spring. On the other hand, the data recorded at Springs D, E, G, H, and I suggest shorter underground residence times since TCC and ICC were mainly recorded in the same orders of magnitude, as expected due to the small catchment sizes. Furthermore, the TCC and ICC patterns recorded at Spring D indicate possible pollution in proximity to near surface conduits and/or swallow holes and estavelles where pollutants could have almost direct access to the aquifer (this also accords with DWK200 information in Table 1), as has been found by Ender et al. (2018).

**Figure 3:**
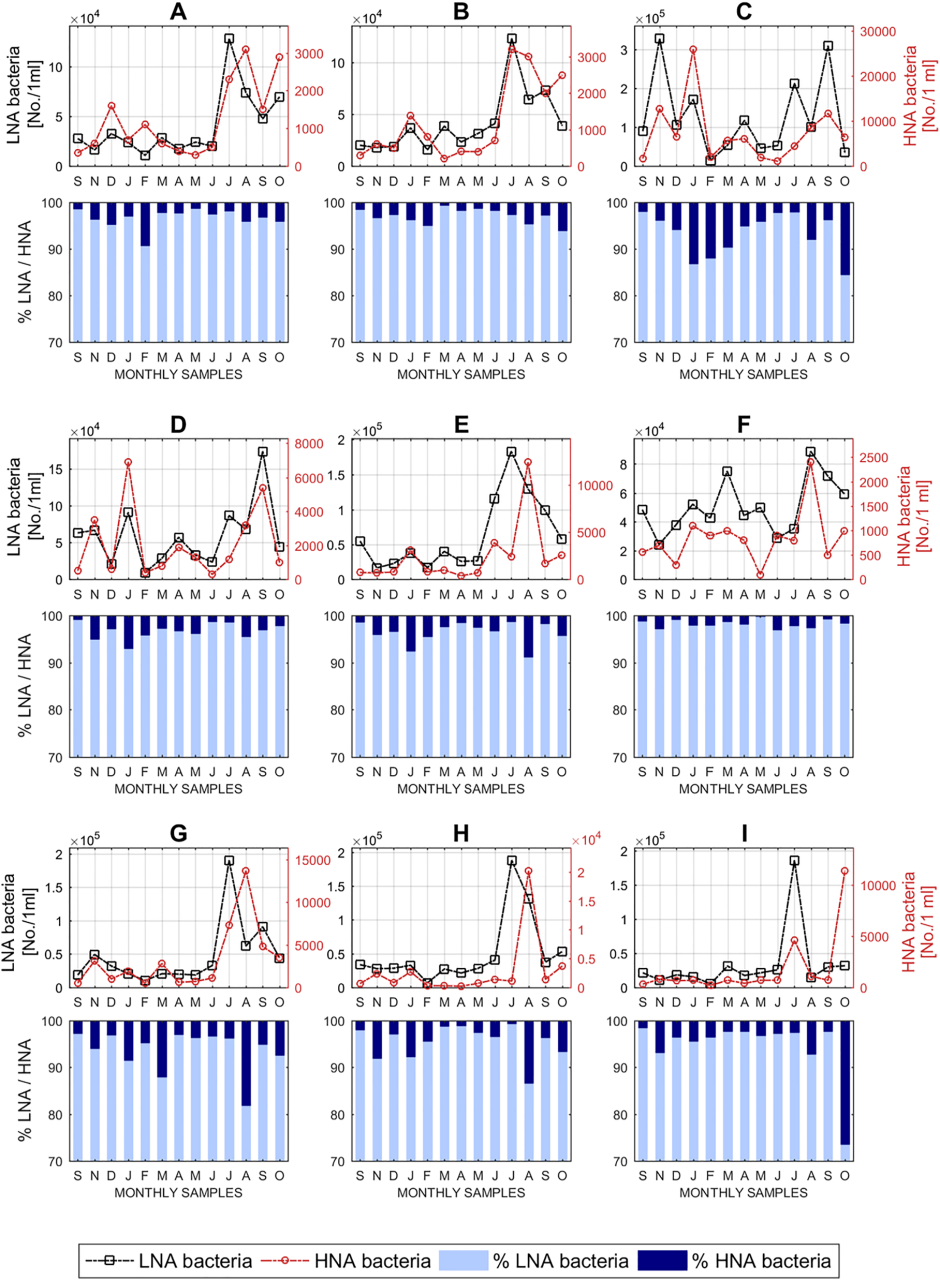
FCM fingerprinting: dynamics of HNA and LNA bacterial populations at karst springs.

Further investigations with HNA and LNA bacterial counts and abundance variations within the ICC groups were then conducted at the karst springs over time (Figure 3).

According to numerous studies of these two distinct fractions of bacteria in other aquatic environments (although predominantly marine), the HNA bacterial populations are considered to be generally larger in size and more active with higher specific metabolic and growth rates in comparison to the LNA bacterial populations (Bouvier et al., 2007). However, not all HNA bacteria may be equally active and some LNA bacteria may be in an active state (Bouvier et al., 2007; Morán et al., 2007). Also, different authors have reported that LNA bacteria ratios were higher in water samples with less contamination (e.g., Salcher et al., 2011; Sharuddin et al., 2018; Santos et al., 2019). Our investigation of nucleic acid groups of bacteria (Figure 3) has revealed that bacteria with LNA characteristics are a highly dominant group of intact bacteria in these karst groundwater samples. However, every karst spring in this study has a distinct FCM fingerprint represented by HNA and LNA concentration variabilities, sudden or gradual count spikes of either nucleic acid group or both at the same time, and relative abundances over time. Since the LNA bacteria are the highly dominant fraction within the ICC cluster in all spring samples, consequently, similar trends between ICC concentration and LNA counts were observed at individual springs. As shown in Figure 3, the percentages of the HNA bacteria rarely tend to rise above 10% within the ICC cluster and only at four karst springs (C, G, H, and I). More elevated HNA percentages were found in situations where samples had very low ICCs within the TCC cluster, for example, in May 2018 at Spring G or in February 2018 at Spring C, and when concentrations of HNA bacteria were significantly higher than usually observed at specific sites. A certain increase in HNA counts can also be seen during summer months especially when groundwater temperatures are higher (Figure 6), providing more favorable environmental conditions for bacteria to survive and remain active longer in the karst systems, as has been previously suggested in in riverine environments (e.g., Santos et al., 2019). However, HNA spikes were detected at Springs C and D even during the winter when groundwater temperatures are notably lower. These results may be explained by faster transport of bacteria in the subsurface to karst aquifer systems, predominantly due to series of larger precipitation events, and increased groundwater velocities, especially through karst conduits.

Furthermore, pH values of groundwater recorded at the karst springs (see Table S1 in Supporting Information S1) were mainly slightly alkaline and near‐neutral alkaline with relatively small variations over time, and hence well within the favorable range for growth and survival of most bacteria, including neutrophilic pathogenic species.

### 3.3 FCM Comparison With Fecal Indicator Bacteria (*E. coli* and Enterococci)

**Figure 4:**
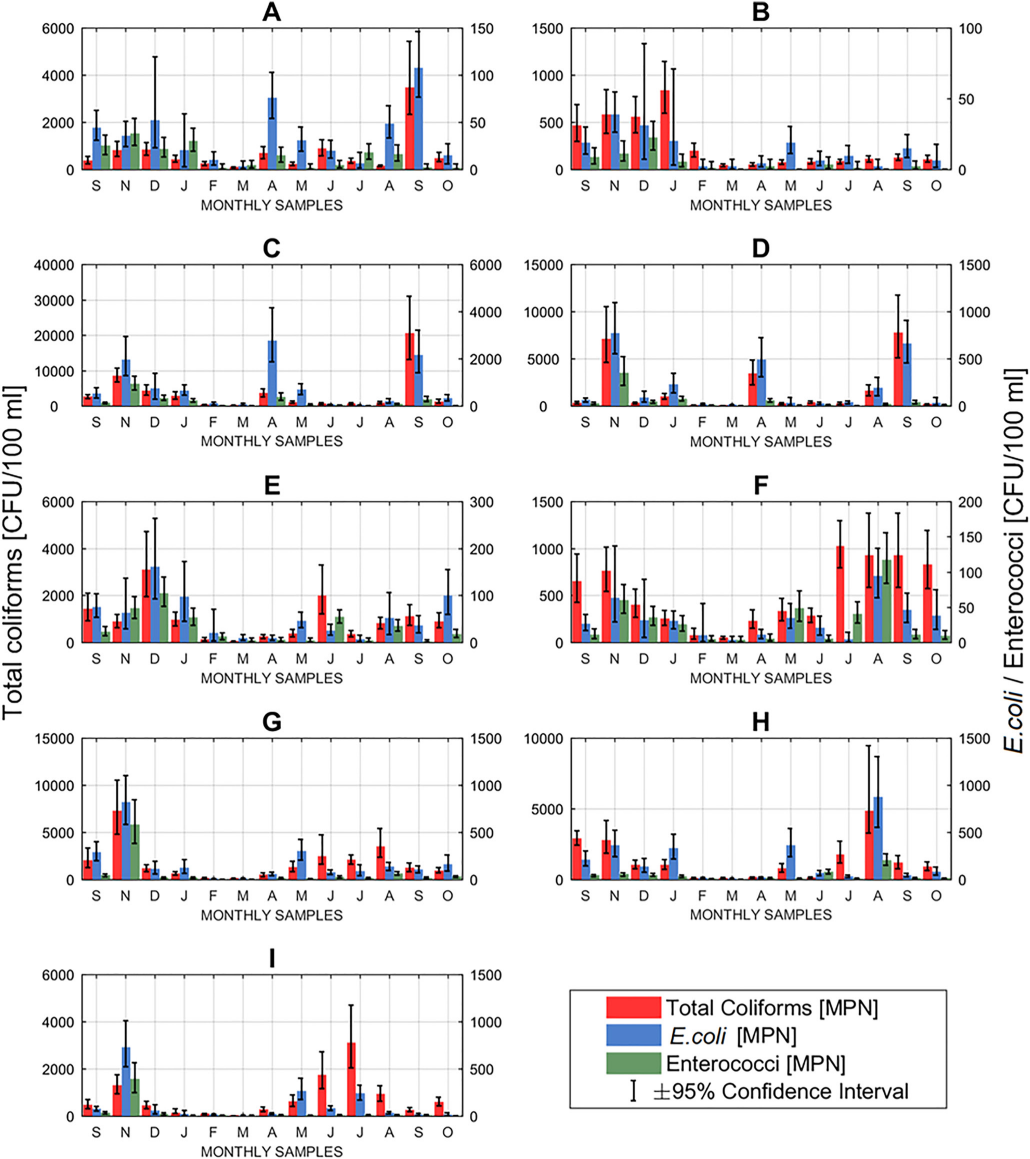
Most probable numbers (MPN) with upper and lower 95% confidence intervals of total coliforms and *E.* *coli* and enterococci found in karst spring samples.

**Figure 5:**
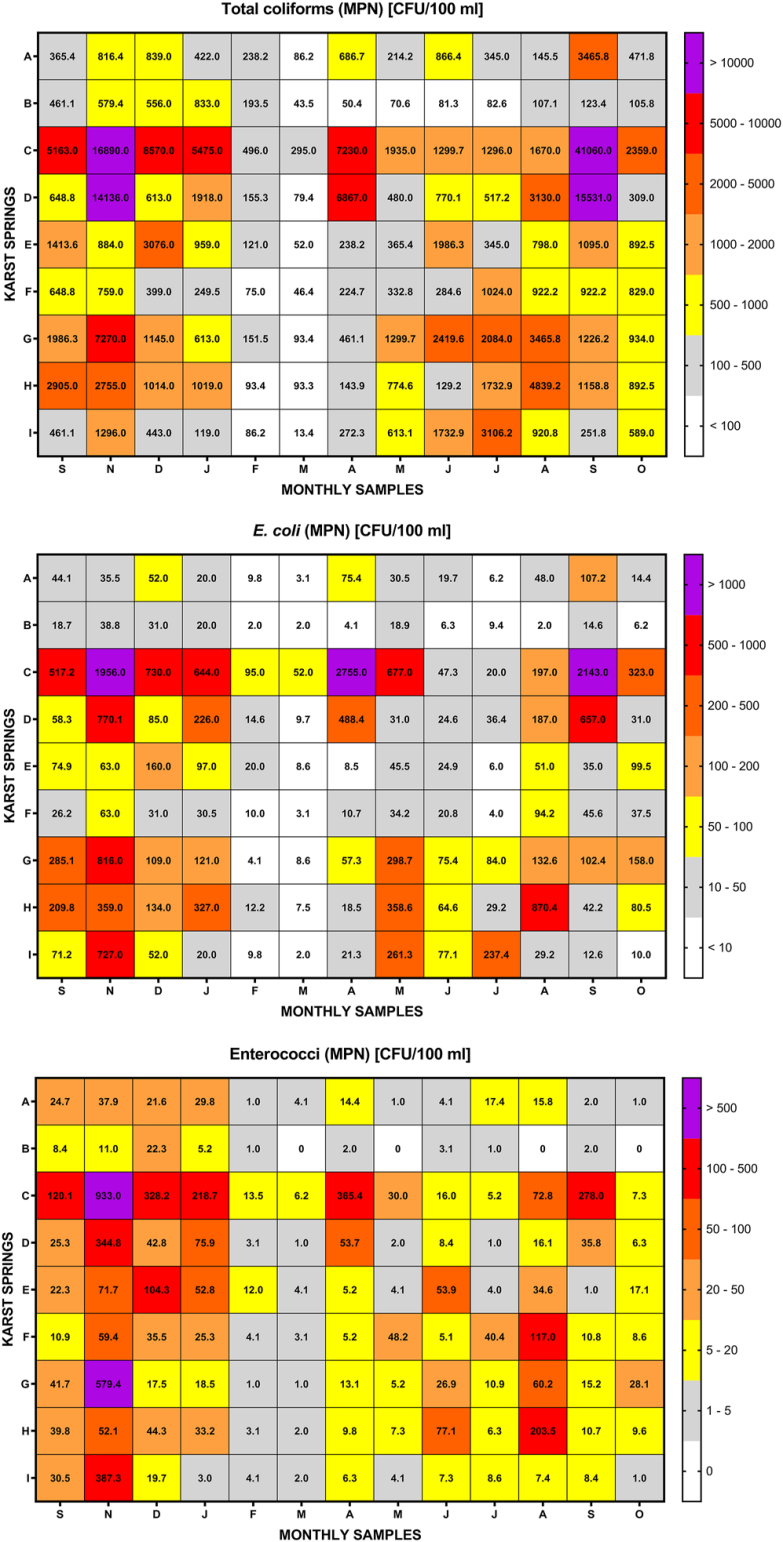
Most probable numbers (MPN) of total coliforms and fecal indicator bacteria (*Escherichia* *coli* and enterococci) found in karst spring samples.

Fecal indicator bacteria (*E. coli* and enterococci) as well as TC counts varied considerably between samples and karst springs as displayed in Figure 4 (which shows the upper and lower 95% confidence intervals of these bacteria) and Figure 5. FIB have been found in all analyzed karst spring samples, indicating considerable fecal pollution of these karst aquifers. Even at Spring I, where significant attenuation of fecal microorganisms might be expected due to the more damped hydrological nature of the karst system with little evident direct infiltration points into the karst aquifer, detected occurrences of FIB are still noteworthy as a result of high densities of DWTSs and agricultural land‐use. Interestingly, this confirms the hypothesis of Sinreich et al. (2014) that moderate TCC values (as observed at Spring I) should be expected for catchments with such contaminant pressures. The highest values of FIB were generally found at Spring C, which was by far the most impacted spring in this study. This is further substantiated by observations based on the FCM data. Spring B is much less impacted by fecal pollution than nearby Spring A, and so it seems clear they are connected to separate karstic systems (which has been suggested previously by Coxon & Drew, 1998, 2000). This is also evident for Springs E and F, where the FIB data further confirms that each is associated with a completely different catchment, despite the fact these springs are emerge within just 50 m of each other, as has been proved by several previous studies using geochemical parameters (e.g., Gill, Babechuk, et al., 2018; Gill, O’Flaherty, et al., 2018). However, it is interesting to highlight that Spring E revealed significantly higher FIB concentrations, in general, in comparison to Spring F which concurs with the previously observed differences in orders of magnitude of ICC values (10^5^ at Spring E and 10^4^ at Spring F). Since TCC values at both springs were measured mostly in the same order of magnitude (10^5^), it shows the additional benefit that the ICC results can bring in complement with the TCC data in terms of understanding microbial pollution of aquifers.

Since the FIB results represent specific live fecal bacterial abundances in the analyzed samples, further investigations have been made to determine whether FCM enumeration of all live bacterial populations (ICC) can be used to predict occurrences of FIB. In addition, other FCM parameters (TCC and HNA) were also investigated with the same objective, with the linear correlations between different FCM and FIB (and TC) variables presented in Table 2.

**Table 2:** Correlation Matrix

Karst spring		FCM TCC	FCM ICC	FCM HNA count	Turbidity
A	Total coliforms	**0.58**	−0.02	0.02	−0.14
	*Escherichia* *coli*	**0.59**	−0.13	−0.03	−0.15
	Enterococci	0.09	−0.05	−0.14	**0.68**
	Turbidity	−0.23	−0.26	−0.15	/
B	Total coliforms	−0.05	−0.37	−0.22	0.87*
	*E. coli*	−0.33	−0.29	−0.30	0.83*
	Enterococci	−0.51	−0.39	−0.36	0.86*
	Turbidity	−0.38	−0.42	−0.34	/
C	Total coliforms	**0.56**	0.75*	0.34	0.34
	*E. coli*	**0.58**	**0.58**	0.28	0.44
	Enterococci	0.93*	0.71*	0.41	0.93*
	Turbidity	0.90*	**0.64**	0.50	/
D	Total coliforms	0.83*	0.69*	**0.58**	**0.60**
	*E. coli*	0.84*	**0.63**	**0.62**	0.71*
	Enterococci	0.70*	0.16	0.37	0.98*
	Turbidity	0.69*	0.17	0.41	/
E	Total coliforms	0.08	−0.01	0.03	0.22
	*E. coli*	−0.33	−0.33	−0.01	0.40
	Enterococci	−0.17	−0.22	0.07	**0.59**
	Turbidity	−0.29	**−0.56**	−0.32	/
F	Total coliforms	−0.23	0.16	0.21	0.16
	*E. coli*	−0.03	0.38	0.50	0.26
	Enterococci	−0.04	0.28	**0.57**	0.16
	Turbidity	0.05	−0.48	−0.38	/
G	Total coliforms	0.76*	0.23	0.33	0.81*
	*E. coli*	**0.57**	−0.04	−0.04	0.87*
	Enterococci	**0.64**	0.02	0.05	0.95*
	Turbidity	**0.60**	0.01	−0.05	/
H	Total coliforms	0.86*	**0.56**	0.77*	0.12
	*E. coli*	0.70*	0.36	0.86*	0.03
	Enterococci	0.77*	0.44	0.91*	0.05
	Turbidity	−0.12	−0.25	−0.15	/
I	Total coliforms	**0.60**	0.80*	0.25	−0.11
	*E. coli*	0.32	0.11	−0.07	0.06
	Enterococci	0.21	−0.14	−0.11	0.14
	Turbidity	−0.03	−0.14	−0.14	/

*Note*. Pearson's correlation coefficient (r) values are bolded in cases where p value is at least <0.05 and bolded with asterisk in cases where p values are <0.01.

It is somewhat surprising that ICC concentrations only show reasonably positive correlations with FIB (TC, *E. coli* and enterococci) numbers at one spring—Spring C—which is by far the most impacted spring by fecal contamination as well as the spring with the highest turbidity levels. Considerably or moderately strong positive correlations were observed between ICC and TC at Springs C, D, H, and I. In addition, a moderate positive correlation has been observed between ICC and *E. coli* at Spring D. However, other strong correlations between ICC and FIB parameters were not found, indicating therefore, that the ICC parameter is not a particularly good indicator of fecal pollution in karst groundwater, especially in less impacted systems where FIB values represent a very small fraction of the entire microbial population. However, the strength of the observed ICC and FIB relationship at Spring C does indicate a possible opportunity for faster and cheaper bacterial monitoring of high pollution events at karst systems, as offered by FCM. Also, it seems clear from the earlier observations and findings that the combination of TCC and ICC may yield very useful insights as it offers a better understanding of microbial dynamics at karst springs and inputs of cell‐rich water from the surface into the subsurface. A somewhat unanticipated finding perhaps were very strong or moderately strong positive correlations between TCC and FIB numbers at several karst springs (C, D, G, and H), exhibiting stronger correlations than those between ICC versus FIB. Furthermore, at Springs A and I, positive correlations were found between TCC and some FIB numbers (TC and/or *E. coli*). This result may be explained by the fact that the ICC concentrations at individual karst springs were relatively stable except during the summer months and low flow conditions, while the TCC and FIB values varied much more throughout the monitoring period (see Figures 2 and 5). Meaningful positive correlation of HNA data with FIB numbers at Spring H probably indicates fecal pollution sources near the spring and is further supported by sharp increases in TCC and ICC data during the summer months pointing to elevated groundwater pollution of agricultural origin from the nearby pastures. Elevated numbers of FIB in groundwater have been found in other pasture‐based land use agricultural karst catchments during the summer season, presumably because domestic animals are out on the field in temperate climates in the summer (e.g., see Mahler et al., 2000).

### 3.4 Turbidity as a Proxy for Microbial Contamination

**Figure 6:**
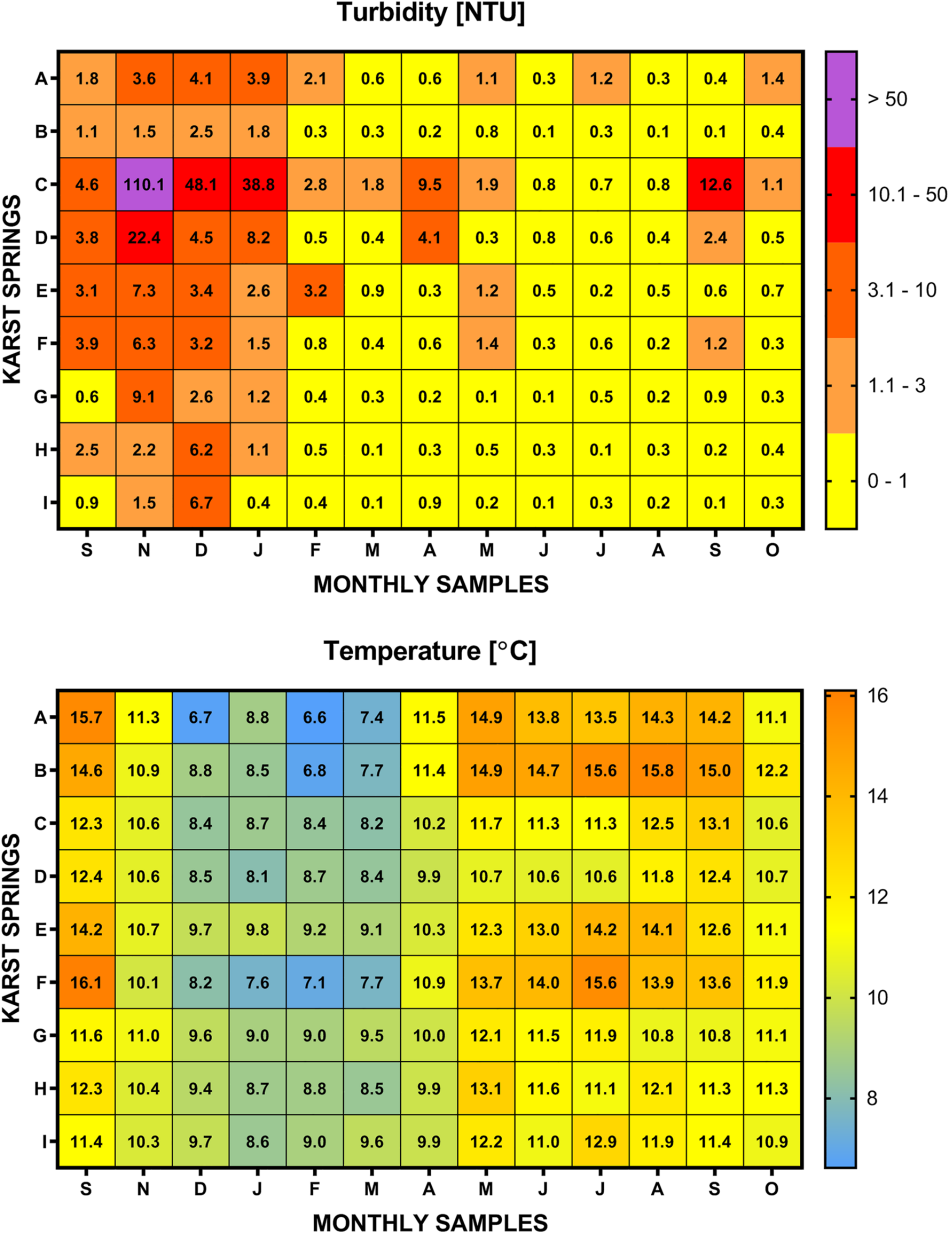
On‐site turbidity and temperature measurements at the time of karst spring sample collections.

The study has also evaluated turbidity (see Figure 6) as a potential inexpensive and more continual indicator of microbial pollution in karst aquifer systems. It was expected that total bacterial concentrations (TCC) would correlate strongly with turbidity. However, the results (Table 2) indicate that turbidity only appears to have a limited potential for predicting general bacterial concentration dynamics, whereby strong and moderate positive correlations were only found at Springs C, D, and G. This can be explained since turbidity is influenced by many factors. For example, as reported by Pronk et al. (2007), the first turbidity pulse at a karst spring may be more associated with the washing out of old settled solids from a karst network (due to piston flow) which is followed by another turbidity pulse associated with the fresh rainfall recharge (and new bacterial contamination) coming through the system. Still, whether the temporal variability of total bacterial densities can be associated with temporal variability of other particulates at individual karst springs is important information, particularly if a spring is being considered as a water supply. More importantly, at six out of nine karst springs, generally strong or moderate positive correlations were found between turbidity and at least one FIB parameter. These findings are not fundamentally different from those found in many earlier studies (e.g., Allen et al., 2008; Huey & Meyer, 2010; Schiperski, 2018) where it has been suggested that turbidity can be used as an inexpensive and easy‐to‐measure indicator of microbial pollution of fecal origin, but systematic relationships are usually highly site‐specific. Interestingly, our results (see Table 2) indicate the strongest correlations between turbidity and enterococci and so perhaps more emphasis in future research could be placed on enterococci alongside the more commonly used bacterial indicators TC and *E. coli* from which insights can then be linked to long‐term continuous monitoring of karst spring turbidity quality.

### 3.5 Influence of Catchment Factors on Microbial Contamination of Karst Springs

The results from the different forms of analysis (FCM, TC, FIB, and turbidity) have been used to assess the influence of different catchment factors (described in Table 1) across the nine spring sites.

The highest average TCC, TC, FIB, and turbidity concentrations were found at Spring C which is one of the smallest catchments. Whilst Catchment C has a relatively low density of DWTS compared to the other study sites, it has one of the higher DWK200 values. Therefore, since its land use and geological setting are not significantly different than those observed at Catchments A, B and C, microbial contamination of Spring C is likely a result of some significant input of contaminants via one or more swallow holes followed by rapid movement of contaminants through the highly transmissive karst network in such a small catchment. After Spring C, the highest TCC and ICC values were found at Springs D, F, and G. Spring D also has the second highest TC, *E. coli* and turbidity values on average. Catchment D is the third largest catchment in this study and has the highest percentage of DWTSs in the catchment that are within 200 m of at least one karst feature (DWK200) that can provide direct input of contaminants to the subsurface; it is also classified as being of Extreme groundwater vulnerability. Similarly, the second highest enterococci concentrations were found at Spring G which has the third highest DWK200 value and is also the smallest catchment among study sites, with the shortest potential contaminant pathways to the spring. On the other hand, the lowest average TC, *E. coli*, enterococci and turbidity values (and among the lowest average TCC and ICC concentrations) were found at Spring B, which is the second largest catchment with relatively low DWTSs density and DWK200 value. Furthermore, the lowest average TCC and ICC values (as well as generally low turbidity) recorded at Spring I can be described mainly due to the nature of the system with no evident direct infiltration points of contaminants, as explained earlier and supported by the hypothesis of Sinreich et al. (2014). It seems that agricultural land use practices during certain periods of the year can explain the sporadic increases of contaminants rather than yielding better insights into the more continuous pollution of the spring.

Finally, the study has revealed that rainfall data is more useful in terms of interpreting the microbial fate and transport through such lowland karst systems, compared to effective rainfall (see Tables S3 and S4 in Supporting Information S1). The relatively fast recharge from the surface into the upper part of the karst system (epikarst), due to thin soil coverage and/or rapid infiltration directly into the groundwater system through discrete flowpaths (swallow holes, etc.) makes such systems less constrained by soil capacity and evapotranspiration processes. This is evidenced by significant FIB occurrences at the springs across the summer months of apparently zero effective rainfall, thereby corroborating the fast arrival of infiltration water. It should also be noted that there were generally higher positive correlations of *E.coli* with rainfall (Table S6 in Supporting Information S1) compared to the TCC correlations with daily rainfall (Table S3 in Supporting Information S1) across all springs, with the peak *E. coli* correlations generally occurring at shorter lag times compared to the TCC results.

## 4 Conclusions

This study has presented the use of flow cytometry as a method for evaluating fecal indicator bacteria across a range of different lowland karst springs over a 14‐month period, as well as evaluating turbidity as a potential real‐time proxy indicator of microbial contamination. This research has shown that flow cytometry can rapidly provide meaningful information about the general levels of bacteria in karst aquifer systems, which can lead to enhanced understanding about the fate and transport of microbial fecal pollution by interpretation of collected data and/or subsequent use of the data for modeling studies. The findings also highlight the importance of FCM analysis in conjunction with other microbiological data (FIB and TC) and physico‐chemical parameters of water in order to reveal more information about fecal pollution sources and pathways in karst catchments. It has been confirmed that TCC is the most useful FCM parameter, although some additional insights can be gained by analyzing ICC and HNA/LNA parameters at the same time. Additionally, this study provides more evidence about existing stable microbial communities in the karst subsurface, this time in lowland karst aquifers in a temperate north‐western Atlantic maritime location. Such apparent stable bacterial communities do, however, dampen the potential of FCM parameters to be able to closely predict changes in FIB at karst springs. The study also shows that further work is needed to compare the quantification of high (HNA) and low (LNA) nucleic acid content bacteria with FCM, in parallel with other more specific and sensitive techniques that can be used for rapid enumeration of fecal bacteria and whether they are in (or out) of a viable but nonculturable (VBNC) state. This is of particular interest in karst systems due to the often rapid transport of such microbial contaminants through the subsurface to a spring. The enumeration of VBNC bacteria in karst groundwater samples together with FCM fingerprinting does not seem to have been investigated to date. This research has also revealed the evident limitations of using turbidity (as an inexpensive means of continuous monitoring) to predict general bacterial concentration dynamics at karst springs. These challenges present opportunities for potential future groundwater quality studies. In particular, more work is needed to fully understand the potential of FCM fingerprinting by conducting more temporally intensive event‐based investigations at karst springs in aquifers where the dynamics of the karst systems have been more fully characterized.

## Supporting information

Supporting Information S1

## Data Availability

Most of the data for this research are included in this paper (and its supplementary information files). The full data sets can be found at this Mendeley Data link: doi: https://doi.org/10.17632/bgn5mcdj69.1.
