# Supplementary material for: Flow Cytometry and Fecal Indicator Bacteria Analyses for Fingerprinting Microbial Pollution in Karst Aquifer Systems
Source: Water Resour Res. 2022 Apr 27;58(5):e2021WR029840. doi: 10.1029/2021WR029840 (PMC9285701; doi:10.1029/2021WR029840)
Supplement: Supplementary file 1 — Supporting Information S1 [file WRCR-58-0-s001.docx]

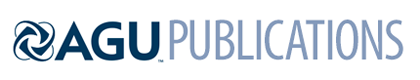


*[Water Resources Research]*

Supporting Information for

**[Flow Cytometry and Faecal Indicator Bacteria Analyses for Fingerprinting Microbial Pollution in Karst Aquifer Systems]**

[Luka Vucinic^1^, David O’Connell^1^, Rui Teixeira^1^, Catherine Coxon^2^ and Laurence Gill^1^]

[^1^Department of Civil, Structural and Environmental Engineering, University of Dublin, Trinity College, Dublin, Ireland

^2^Department of Geology and Trinity Centre for the Environment, University of Dublin, Trinity College, Dublin, Ireland]

**Contents of this file**

Figures S1 to S2

Tables S1 to S6

**Introduction**

The following figures and tables provide additional information for inferences made in the main text of the manuscript.


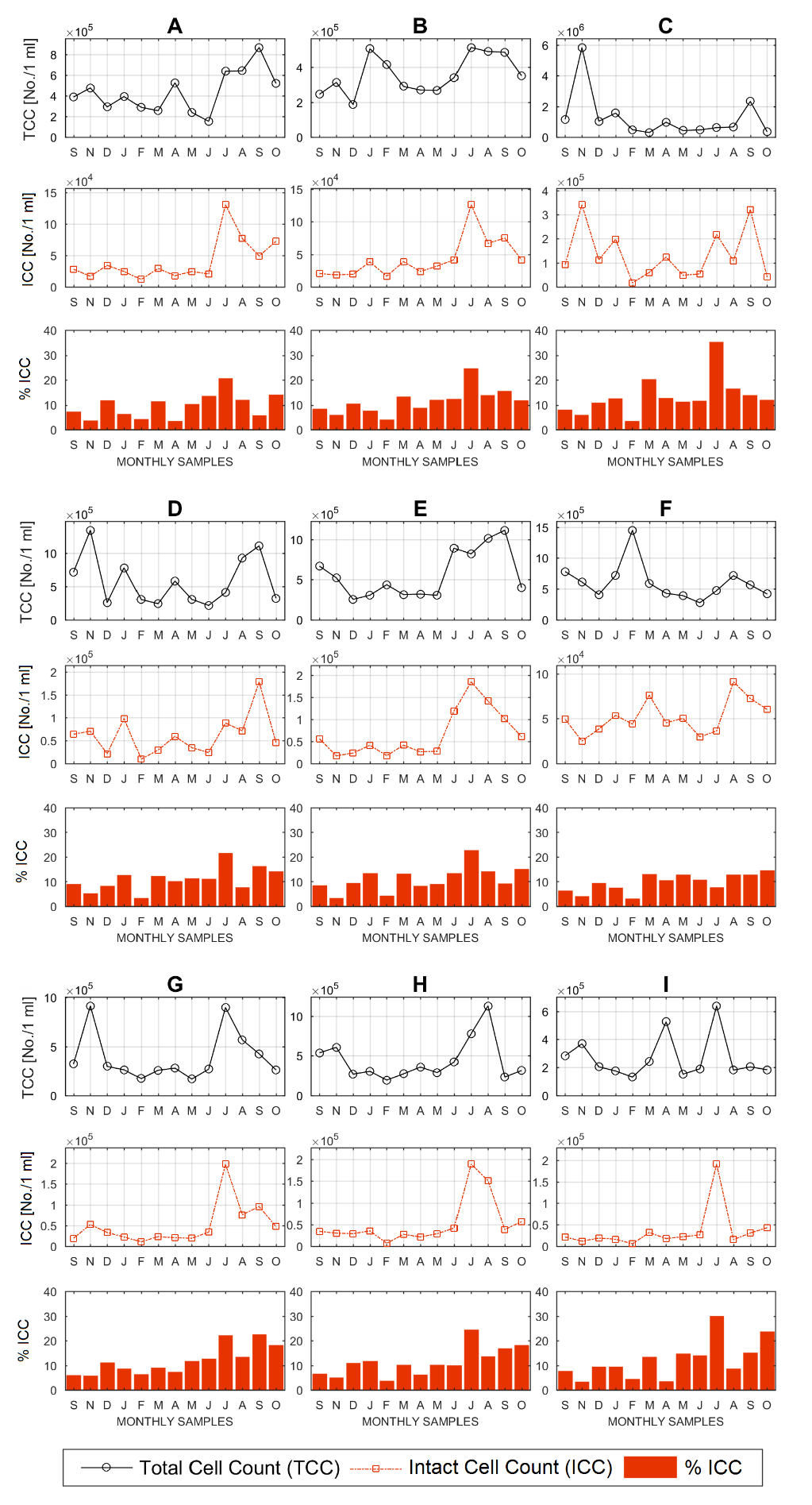


**Figure S1.** FCM fingerprinting: TCC, ICC and %ICC


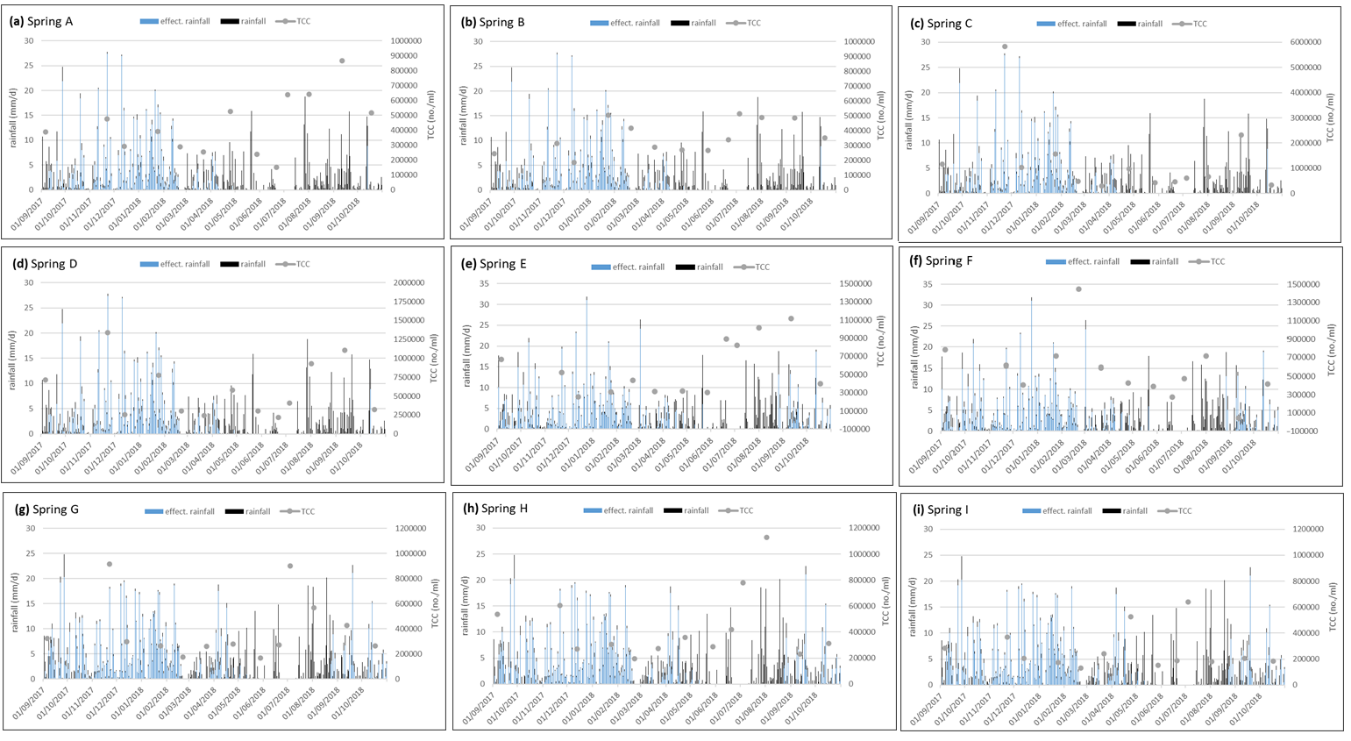


**Figure S2.** Rainfall, effective rainfall and Total Cell Counts (TCC) from flow cytometry against time for the 9 karst spring sites.

| **Table S1.** pH data | | | | | | | | | | | | | | |
| --- | --- | --- | --- | --- | --- | --- | --- | --- | --- | --- | --- | --- | --- | --- |
| **Spring** | Sep. 2017 | Nov. 2017 | Dec. 2017 | Jan. 2018 | Feb. 2018 | Mar. 2018 | Apr. 2018 | May 2018 | Jun. 2018 | Jul.  2018 | Aug. 2018 | Sep. 2018 | Oct. 2018 |  |
| **A** | 7.60 | 6.94 | 7.28 | 7.40 | 7.88 | 8.25 | 7.69 | 7.80 | 7.70 | 7.65 | 7.51 | 7.59 | 7.88 |  |
| **B** | 7.72 | 7.19 | 7.51 | 7.68 | 7.75 | 8.05 | 7.64 | 7.68 | 7.57 | 7.74 | 7.68 | 7.88 | 7.76 |  |
| **C** | 7.55 | 7.34 | 7.47 | 7.44 | 7.43 | 7.72 | 7.58 | 7.28 | 7.15 | 7.24 | 7.31 | 7.55 | 7.43 |  |
| **D** | 7.11 | 7.36 | 7.54 | 7.91 | 7.58 | 7.81 | 7.84 | 7.97 | 7.58 | 7.43 | 7.61 | 7.83 | 7.67 |  |
| **E** | 7.58 | 6.98 | 6.84 | 7.25 | 7.33 | 7.31 | 7.34 | 7.19 | 7.32 | 7.37 | 7.38 | 7.48 | 7.53 |  |
| **F** | 7.65 | 7.42 | 7.46 | 7.73 | 7.78 | 7.64 | 7.51 | 7.52 | 7.52 | 7.70 | 7.59 | 7.77 | 7.93 |  |
| **G** | 7.36 | 6.98 | 7.15 | 7.11 | 7.23 | 7.56 | 7.16 | 7.08 | 7.22 | 7.47 | 7.38 | 7.28 | 7.41 |  |
| **H** | 7.17 | 7.14 | 7.12 | 7.22 | 7.26 | 7.32 | 7.66 | 7.11 | 7.14 | 7.54 | 7.16 | 7.29 | 7.18 |  |
| **I** | 7.16 | 7.10 | 7.14 | 6.95 | 7.18 | 7.25 | 6.94 | 7.09 | 7.11 | 7.09 | 7.18 | 7.31 | 7.09 |  |

| **Table S2**. Electrical conductivity data (µS/cm) | | | | | | | | | | | | | |
| --- | --- | --- | --- | --- | --- | --- | --- | --- | --- | --- | --- | --- | --- |
| **Spring** | Sep. 2017 | Nov. 2017 | Dec. 2017 | Jan. 2018 | Feb. 2018 | Mar. 2018 | Apr. 2018 | May 2018 | Jun. 2018 | Jul.  2018 | Aug. 2018 | Sep. 2018 | Oct. 2018 |
| **A** | 529 | 477 | 441 | 336 | 305 | 335 | 354 | 385 | 402 | 371 | 401 | 392 | 359 |
| **B** | 442 | 488 | 403 | 312 | 278 | 285 | 310 | 318 | 314 | 309 | 336 | 303 | 292 |
| **C** | 481 | 220 | 286 | 219 | 328 | 338 | 281 | 406 | 472 | 480 | 598 | 271 | 333 |
| **D** | 563 | 280 | 326 | 175 | 271 | 298 | 257 | 332 | 368 | 396 | 292 | 292 | 309 |
| **E** | 589 | 561 | 685 | 453 | 431 | 433 | 436 | 410 | 4530 | 26260 | 6084 | 675 | 378 |
| **F** | 438 | 243 | 345 | 273 | 237 | 258 | 258 | 654 | 15830 | 12110 | 8610 | 2328 | 793 |
| **G** | 773 | 653 | 638 | 456 | 488 | 530 | 499 | 558 | 568 | 588 | 552 | 544 | 535 |
| **H** | 730 | 619 | 611 | 437 | 451 | 399 | 442 | 470 | 512 | 512 | 508 | 530 | 512 |
| **I** | 795 | 666 | 654 | 478 | 501 | 545 | 524 | 572 | 560 | 596 | 577 | 569 | 564 |

**Table S3.** Correlation coefficients between TCC counts at springs and lagged daily rainfall and antecedent cumulative rainfall with respect to the day of sampling.*

^α^ day = daily rainfall on the day of sampling; -1 day = daily rainfall on day before sampling; -2 day = daily rainfall 2 days before sampling etc.

^β^ 1 day = antecedent cumulative rainfall on day of sampling and day before sampling; 2 day = antecedent cumulative rainfall back to 2 days before sampling etc.

**Table S4.** Correlation coefficients between TCC counts at springs and lagged daily effective rainfall and antecedent cumulative effective rainfall with respect to the day of sampling.*

^α^ day = daily rainfall on the day of sampling; -1 day = daily rainfall on day before sampling; -2 day = daily rainfall 2 days before sampling etc.

^β^ 1 day = antecedent cumulative rainfall on day of sampling and day before sampling; 2 day = antecedent cumulative rainfall back to 2 days before sampling etc.

**Table S5.** Correlation coefficients between total coliforms at springs and lagged daily rainfall and antecedent cumulative rainfall with respect to the day of sampling.*

^α^ day = daily rainfall on the day of sampling; -1 day = daily rainfall on day before sampling; -2 day = daily rainfall 2 days before sampling etc.

^β^ 1 day = antecedent cumulative rainfall on day of sampling and day before sampling; 2 day = antecedent cumulative rainfall back to 2 days before sampling etc.

**Table S6.** Correlation coefficients between *E. coli* at springs and lagged daily rainfall and antecedent cumulative rainfall with respect to the day of sampling.*

^α^ day = daily rainfall on the day of sampling; -1 day = daily rainfall on day before sampling; -2 day = daily rainfall 2 days before sampling etc.

^β^ 1 day = antecedent cumulative rainfall on day of sampling and day before sampling; 2 day = antecedent cumulative rainfall back to 2 days before sampling etc.

* yellow and green highlights in Tables S3 - S6 were used to show the highest correlation for each spring
